# The Architect of Neurotransmission in *C. elegans* : How FLP-3 Neuropeptides' Structures Direct their Function

**DOI:** 10.17912/micropub.biology.000829

**Published:** 2023-05-27

**Authors:** Rehab Salama, Elizabeth DiLoreto, Jagan Srinivasan

**Affiliations:** 1 Biology and Biotechnology, Worcester Polytechnic Institute; 2 Neuroscience Program, Worcester Polytechnic Institute

## Abstract

Neuropeptides direct functions in the nervous, endocrine, and immune systems of all animals by altering the activity at neural synapses. A single neuropeptide gene can be post-translationally modified to create multiple active peptides. These individual active peptides can have unique functions and drive discrete binding partners. We have previously shown that specific peptides encoded by the *C. elegans* neuropeptide gene, *flp-* 3, have sex-specific roles in response to a pheromone released by hermaphrodite *C. elegans,* ascaroside #8 (ascr#8). Using structural predictions of select FLP-3 neuropeptides, we identify individual amino acids within specific neuropeptides that regulate specific behaviors suggesting structure-function relationships of neuropeptides in regulate sex-specific behaviors.

**Figure 1. Neuropeptide conformation impacts behavioral response to ascr#8 detection in male loss of flp-3 function worms:**
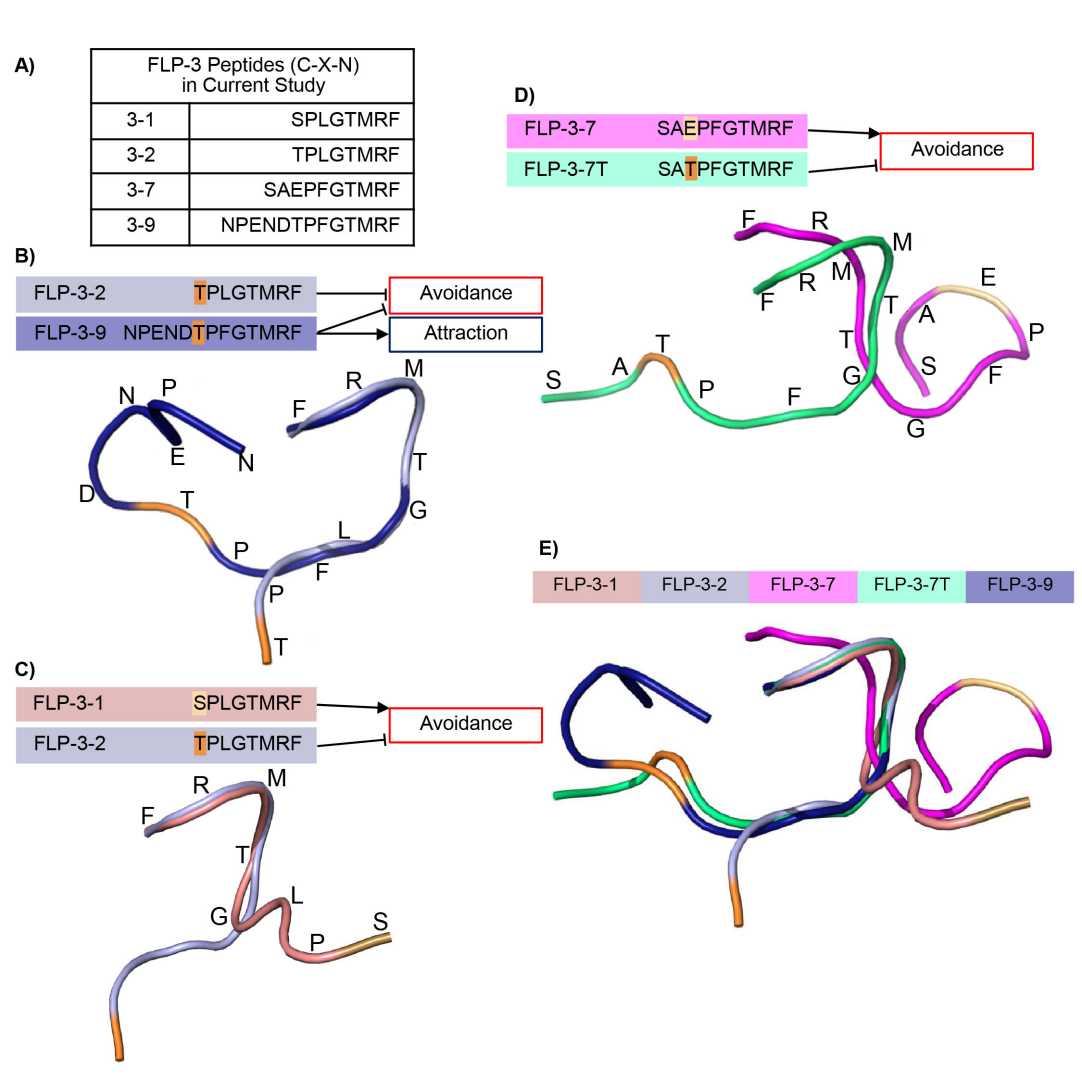
**A)** Peptides produced by the *flp-3* gene presented in the current study. **B)** FLP-3-2 (light blue) and the threonine in position 8 (T8) from the N-terminus (orange) inhibits avoidance, FLP-3-9 (dark blue) and T8 (orange) inhibits avoidance and promoted attraction. **C)** FLP-3-1 (rose) promoted avoidance while FLP-3-2 (light blue) inhibits avoidance, both contain the first 7 amino acids except for a serine in position 8 for FLP-3-1 (tan). **D)** FLP-3-7 (magenta) and E8 (tan) promotes avoidance, FLP-3-7T (cyan) and T8 (orange) inhibits avoidance. **E)** FLP-3-7T superimposed with, FLP-3-1, FLP-3-2, FLP-3-7,and FLP-3-9.

## Description

Ascarosides are modular sugar derivatives produced by *C. elegans* for communication within their species. They are composed of 4 parts; head group, sugar, lipid side chain, and terminal group, with different moieties that produce distinct functions (Muirhead & Srinivasan, 2020) . Previous work found that wildtype hermaphrodites secrete ascr#8, which attracts male worms and repels other hermaphrodites (Narayan et al., 2016; Reilly et al., 2021) . Our group further uncovered that loss of function in the *flp-3* gene causes male worms to avoid ascr#8 rather than find it attractive (Reilly et al., 2021) . The *flp-3* gene produces ten discrete FLP-3 peptides named FLP-3-1 to FLP-3-10. **Figure 1A** highlights the FLP-3 peptides discussed in the current study. In prior studies, rescue of individual neuropeptides was accomplished by a peptide feeding approach where genetic information to produce a single neuropeptide was encoded in a vector which was transformed into *E. coli* bacteria that *C. elegans* consumed (Reilly et al., 2021) . These bacteria encoding individual peptides were fed to *flp-3* loss of function worms. Any peptide activity was directed by the exogenous feeding of the bacterially-encoded individual peptides. Previous studies from our lab showed that restoration of FLP-3-2 or FLP-3-9 peptide caused loss of basal ascr#8 avoidance in the *flp-3* mutant worms, **Figure 1B** (Reilly et al., 2021) . FLP-3-2 caused a loss of avoidance, yet there was no gain of attraction towards ascr#8, leaving the male worms neutral towards the ascaroside. Additionally, FLP-3-9 was sufficient to restore native male attraction to ascr#8, suggesting that FLP-3-9 is necessary for male worms to find ascr#8 and therefore hermaphrodites attractive. The difference in behavior associated with individual FLP-3 peptide rescue suggests that the neuropeptides’ role in ascr#8 attraction is related to peptide structure.

Our lab’s previous behavioral work investigating the relationship between the FLP-3 peptides and ascr#8 responses spurs our current work to model the structures of the FLP-3 neuropeptides and determine the role of the peptides’ sequences and related structures. To determine any structure-function relationships between the rescuing peptides, we predicted the conformational structures of FLP-3 neuropeptides using PEP-FOLD3 (Lamiable et al., 2016) . We had particular interest in peptides FLP-3-2 and FLP-3-9 that resulted in behavioral changes in male *flp-3* loss of function worms in a previous study (Reilly et al., 2021) . To assess for structural similarity between the peptides, we optimized confirmations and then aligned them with one another along the α-carbon, starting at the amide end of the peptide by PyMol. Alignment of the predicted structures of FLP-3-2 (light blue) and FLP-3-9 (dark blue) suggests that they share the conformation structure with RMSD = 0.224, **Figure 1B** . FLP-3-2 and FLP-3-9 share the same sequence of the first eight N- terminal amino acids, with the replacement of lysine in FLP-3-2 with phenylalanine in FLP-3-9 at position six, suggesting this region may be connected with the loss of avoidance behavior seen when feeding occurred with these individual peptides. The five extra residues that FLP-3-9 has may be responsible for attraction gain.

Reilly *et al.* 2021 suggested that the threonine closest to the C-terminus is critical for eliminating ascr#8 avoidance. We investigated the importance of this threonine by comparing other FLP-3 peptides that do not have this amino acid. FLP-3-1 (rose) has the same structure as FLP-3-2 (light blue) with serine instead of threonine. Previously it was shown that the uptake of FLP-3-1 did not change the avoidance behavior to ascr#8 in *flp-3* loss of function worms (Reilly et al., 2021) , **Figure 1C** . When we aligned the structures of FLP-3-1 and FLP-3-2, we found that there was divergent alignment starting at the glycine residue, despite the fact that the only difference is the C-terminal amino acid. FLP-3-1 and FLP-3-2 aligned with RMSD = 0.231. FLP-3-7 also has a similar sequence, except with a glutamate at position eight rather than the threonine. Earlier investigation showed that when FLP-3-7 was fed to *flp-3* loss of function male worms, they still avoided ascr#8, **Figure 1D .** Previous behavioral work by Reilly *et al.* modified the vector encoding FLP-3-7 experimentally to produce a mutated version with threonine replacing the glutamate. The FLP-3-7T peptide was fed to *flp-3* loss of function male worms, resulting in non-avoidance to ascr#8. In contrast this peptide did not exhibit wildtype attraction to ascr#8. In our current work, we found that superimposing the structures of FLP-3-7 and FLP-3-7T shows that FLP-3-7 (magenta) changes conformation when a threonine is present in position eight, FLP-3-7T (teal), with RMSD = 2.323, **Figure 1D .** Compared to the original FLP-3 peptides tested, FLP-3-7 has a different conformation than FLP-3-2 and FLP-3-9 with RMSD = 2.350 and 2.387, respectively, **Figure 1E** . When we changed the glutamate at position eight from the N-terminus to a threonine (FLP-3-7T), the altered peptide conformation was similar to FLP-3-2 and FLP-3-9 with RMSD = 0.075, 0.208 respectively **Figure 1E** .

Overall, our computational results suggest a structure-function relationship among neuropeptides, influenced by single amino acid substitutions. Of the peptides discussed here, FLP-3-1, FLP-3-2, FLP-3-7, FLP-3-9, all are predicted to bind to target receptors NPR-10 and FRPR-16. All the peptides have a stronger affinity for FRPR-16 over NPR-10. Additionally, FLP-3-1 has the highest affinity for each receptor and FLP-3-9 the lowest (Reilly et al., 2021) . The differences in receptor binding affinity to individual FLP-3 peptides suggest that changes in behavior in response to ascr#8 is directed by single amino acid changes to these neuropeptides. This comes while considering that the peptide FLP-3-9 is the sole FLP-3 peptide that overrides the basal ascr#8 avoidance and restores male’s attraction to hermaphrodite released ascr#8 in the loss of *flp-3* function worms, yet it has the lowest binding affinity to NPR-10 or FRPR-16. The structural alignments presented in this work corroborates our previously described behavioral data (Reilly et al., 2021) further confirming the role of FLP-3 peptides, especially FLP-3-9, in regulating male *C. elegans* attraction to hermaphrodite pheromone ascr#8. Our studies identify molecular-level conformational changes in amino acids strongly correlate to the overall function of the peptide suggesting that the dynamics of amino acids within a peptide/protein can dictate the overall function and activity.

## Methods

The PEP-FOLD3 *de novo* technique (Lamiable et al., 2016; Maupetit, Derreumaux, & Tuffery, 2009; Maupetit, Derreumaux, & Tufféry, 2010; Shen, Maupetit, Derreumaux, & Tuffery, 2014; Thévenet et al., 2012) was used to predict the conformational tertiary structure of the neuropeptides. This platform is used for sequence predictions between 5-50 amino acids, a range in which most neuropeptides fit. Once the initial peptide sequences are inserted, the standard free modeling parameters were used. After running a short molecular dynamic simulation, the platform generates 100 models, sorts the clusters using sOPEP energy value or Apollo predicted TMscore, and ranks the top five models. The structure with the highest sOPEP value was chosen to represent the native or near native conformation of the neuropeptides comparison. FLP-3-1, FLP-3-2, FLP-3-7, FLP-3-7T, and FLP-3-9 predicted structures were superimposed by the α carbon of residues 1 to 5 by PyMOL. The root mean square deviation (RMSD) reflects the square root of the average of the square of the distances between the complement atoms (Schrödinger).

## Reagents

N/A
